# Research Electronic Data Capture (REDCap) for Population-Based Data Collection in Low- and Middle-Income Countries: Opportunities, Challenges, and Solutions

**DOI:** 10.2196/65377

**Published:** 2025-06-11

**Authors:** Ha Thanh Le, Dung Viet Tien Vu, Thi Ngoc Anh Nguyen, Hang Tran Thi, Tan Viet Nguyen, Thao Phuong Tran, Aria Kekalih, Samita Rijal, Dewi Friska, Raph L. Hamers, Abhilasha Karkey, Mary Chambers, Jennifer Ilo Van Nuil, SPEAR and CoAct Team, Sonia Lewycka

**Affiliations:** 1 Oxford University Clinical Research Unit Hanoi Vietnam; 2 Community Medicine Department Faculty of Medicine Universitas Indonesia Jakarta Indonesia; 3 Oxford University Clinical Research Unit Kathmandu Nepal; 4 Faculty of Medicine Universitas Indonesia Oxford University Clinical Research Unit Indonesia Jakarta Indonesia; 5 Nuffield Department of Medicine Centre for Tropical Medicine and Global Health University of Oxford Oxford United Kingdom; 6 Oxford University Clinical Research Unit Ho Chi Minh Vietnam; 7 See Acknowledgments

**Keywords:** REDCap, population-based research, data collection, data management, strength, limitation.

## Abstract

Health research requires high-quality data, and population-based health research comes with specific opportunities and challenges for data collection. Electronic data capture can mitigate some of the challenges of working with large populations in multiple, sometimes difficult-to-reach, locations. This viewpoint paper aims to describe experiences during the implementation of two mixed methods studies in Vietnam, Nepal, and Indonesia, focusing on understanding lived experiences of the COVID-19 pandemic across 3 countries and understanding knowledge and behaviors related to antibiotic use in Vietnam. We present the opportunities, challenges, and solutions arising through using Research Electronic Data Capture (REDCap) for designing, collecting, and managing data. Electronic data capture using REDCap made it possible to collect data from large populations in different settings. Challenges related to working in multiple languages, unstable internet connections, and complex questionnaires with nested forms. Some data collectors lacked the digital skills to comfortably use REDCap. To overcome these challenges, we included regular team meetings, training, supervision, and automated error-checking procedures. The main types of errors that remained were incomplete and duplicate records due to disruption during data collection. However, with immediate access to data, we were able to identify and troubleshoot these problems quickly, while data collection was still in progress. By detailing our lessons learned—such as the importance of iterative testing, regular intersite meetings, and customized modifications—we provide a roadmap for future projects to boost productivity, enhance data quality, and effectively conduct large-scale population-based research. Our suggestions will be beneficial for research teams working with electronic data capture for population-based data.

## Opportunities Offered by Electronic Data Capture

Research quality is highly dependent on appropriate design, methodology, and data collection [1]. An important component of public health research quality is data accuracy and reliability [2]. Growing international research, increased complexity of study designs, and stricter legislation of sharing health data, including ethics, privacy, and data infrastructure, make systems for high-quality data collection, management, and storage more crucial, but also more challenging than ever [3,4]. Electronic data collection methods can reduce common data entry errors and enhance data collection, storage, and analysis, compared to traditional tools [5,6]. However, when planning research, investigators need to weigh the trade-offs and benefits of different approaches.

Population-based data are essential in epidemiology for understanding health issues and determinants and informing public health decisions and policies. Compared with clinical research, which uses patient records at health care facilities, population-based research relies on surveys of large, diverse, and representative community samples, often requiring researchers to locate and interview participants in the field.

Poor quality data collection undermines population data usefulness and analysis validity, with quality issues arising at every stage of the research process [7]. Paper-based questionnaires remain common in population-based research, but electronic data capture offers real-time data management, enhanced fieldwork efficiency, and improved integration of collection, management, and security.

## Data Collection

For both paper-based and electronic data capture, data collection tools must be well-designed and data collectors well-trained in order to minimize incorrect, biased, or missing data. Errors can happen at different times during data collection and in almost all research, even in a well-designed and controlled study [8,9]. A study developing the data error criteria for retrospective studies analyzed 16 publications and identified 2515 general errors, of which 1920 (18.9%) were blank cells, 556 (5.5%) were caused by spreadsheet mismanagement, 32 (0.3%) by transcribing errors, and 7 (0.1%) were coder-related [10]. In light of this, some studies have concentrated on how to handle missing data, issues caused by missing data, and the methods to prevent or minimize missing data in medical research [11,12]. Rigorous training for everyone involved in the study before participant enrollment begins is an important step to minimize errors and should include everything from enrolling participants to detailed instructions and practice for data collection and data entry processes [3]. Electronic data capture can also reduce errors at the point of data collection and entry.

Paper-based data collection has the advantage that no technical or IT skills are needed to develop forms or enter data, no hardware or software is needed, and no internet connection is needed to upload data to a central server. This makes tools accessible in remote settings. However, the costs and resources required for printing and distributing questionnaires may be considerable, and it is easy for data collectors to make mistakes with branching logic or record unrealistic values. In contrast, electronic data capture can save time with data entry. Field restrictions and branching logic may reduce errors at the point of entry, allowing fields to be concealed or shown depending on responses to previous survey items. Data collection instruments can be shorter and more logical if related questions are grouped and only presented to respondents when relevant. Furthermore, self-completed surveys can be shared en masse using survey links, providing a cost-effective solution for large-scale survey deployment across countries and regions [13]. However, a basic level of IT skills is required by data collectors or participants, which may limit access for some populations (eg, those with low IT literacy) [6,14].

## Data Management

Large-scale studies have distinct advantages in etiology research because of large sample sizes and increased statistical power, but face challenges in terms of data management and quality control. Population-based studies use a range of data collection tools with different features administered by different data collectors and at different times. Hence, research design must take into account the study needs, team skills, and implementation logistics [15]. Recognizing these challenges, funders and institutes are increasingly requesting researchers to develop data management plans before, during, and after data collection. These plans must outline how each step will be monitored so that it is possible to keep track of what happens to the data [16,17]. The advantage of paper-based data collection for data management is that a physical record of the interview is retained and can be used to verify data in the system and correct possible data entry errors. The main disadvantage is the time taken to enter and check large volumes of data. Electronic data capture allows data to be stored immediately, making it available for real-time checking and callbacks to participants to correct errors. It provides researchers and data managers with an overview of the dataset, even for a single variable, at any time, allowing them to keep control over data quality. Electronic data capture also enables simultaneous data collection and management in multiple languages using a single tool and database, which is an advantage for large multi-country surveys. Furthermore, many data capture platforms have integrated functionality that allows automatic reports to be generated in real time, as data collection is ongoing. While electronic data capture (EDC) systems are advantageous, they need greater investment when compared with paper-based systems in terms of upfront costs, such as infrastructure and training. These expenses cover purchasing equipment, software licenses, secure storage systems, and training staff on how to use the tools. On the other hand, paper-based systems are initially less expensive but may eventually increase costs because of sluggish data processing, manpower requirements, and the need to correct potential mistakes. Choosing the best data collection strategy requires researchers to thoroughly evaluate the balance between the cost and data quality.

## Data Storage and Sharing

There are many different rules and guidelines regarding how long data and records should be maintained, since longer-term and permanent retention of research outputs is necessary as long as possible after publication [18,19]. When working with large amounts of data, data storage capacity may be a challenge. Paper-based records may be bulky, especially for large surveys, and require adequate physical space to retain them. They must be protected from damage by water, insects, and fire. Well-organized and maintained filing systems are required to easily retrieve specific documents. Electronic data capture allows data to be immediately uploaded to a central server, where regular backups can be made to avoid data loss. However, sufficient data storage capacity may be needed for large surveys. In order to strengthen scientific transparency and increase the value-for-money of data collected, it is increasingly recommended by journals and research funding institutions to share data and project documents as widely as possible. Fortunately, there are several features of electronic data collection systems that can automatically create data dictionaries, codebooks, and forms, which facilitate the understanding and interpretability of the data and the research project in general [16].

## Security

Research ethics incorporate the ideals of beneficence, justice, protection of an individual’s identity, and dignity [20,21]. Data must be processed in a manner that ensures the proper confidentiality of personal and identifiable information, including protection against unintentional loss, destruction, or damage, using appropriate technical or organizational measures [22,23]. When it is absolutely necessary to be kept in a data file, personal data should be encrypted [24]. Research needs secure data management tools or systems that can tackle this issue. Hard copies of data should be stored in locked rooms or cupboards that are only accessible by the study team. Access to these is easily restricted, and it is unlikely that copies of physical documents will be inadvertently made and shared. Electronic data, likewise, should only be accessible by authorized members of the study team. Institutions should use a user authentication system and an advanced encryption algorithm to ensure that data remains secure. It may be difficult to ensure data security for data collected with traditional tools such as paper or spreadsheets [25]. In the building and developing process, electronic data collection systems are designed to be consistent with different information security standards worldwide, which have some features to improve the procedures for setting up secure data collection, including the ability to set specific user privileges, create data access groups, and lock records [26].

## Overview of Research Electronic Data Capture and Comparable Tools for Data Collection

The REDCap (Research Electronic Data Capture) platform is a web-based application for building and managing web-based databases and surveys. REDCap supports several types of research, such as cross-sectional studies, clinical trials, retrospective studies, cohort studies, and many more [26,27]. The usability of REDCap goes beyond data collection because it includes a number of tools that are advantageous in the research environment, such as interview scheduling and customized data analysis. It is also freely available, making it a good option for research centers to use while conducting epidemiological investigations [6]. REDCap is compliant with FISMA (Federal Information Security Management Act), GDPR (General Data Protection Regulation), HIPAA (Health Insurance Portability and Accountability Act), and 21 CFR Part 11 (Part 11 of Title 21 of the Code of Federal Regulations) [13]. While REDCap is ideal for academic research that requires advanced customization and regulatory compliance, Open Data Kit (ODK) is better suited for fieldwork in remote settings, as it is optimized for offline data collection and is open-source, making it lightweight and customizable [28]. Other platforms, such as KoboToolbox and KoBo Collect, expand ODK’s functionality by making the platform more user-friendly, secure, and feature-rich [29,30]. In contrast, commercial tools like Qualtrics offer advanced prebuilt tools with sophisticated analytics and survey design capabilities for a broader audience, including academic and enterprise-level needs [31]. With 7231 consortium partners spread across 156 countries and over 39,000 citations (information updated in May 2024), REDCap remains a widely adopted and trusted platform in the global research community [32]. As a result, the academic community commonly uses this tool for ongoing research initiatives, ensuring a safer environment for research outcomes.

This article provides an overview of experiences using REDCap in 2 multisite population-based studies: Social Science and Public Engagement Action Research (SPEAR) and Collective Action against antimicrobial resistance in Vietnam (CoAct). The aim of this article is to describe experiences during the implementation of 2 mixed methods studies and discuss the opportunities, challenges, and solutions arising through using REDCap for designing, collecting, and managing data. The lessons learned will be beneficial to research teams conducting population-based health research, as well as software development teams aiming to improve effective data management in the field.

## Project Settings

### The SPEAR Project

The SPEAR study was a mixed methods social science study, combining surveys, in-depth interviews, and social media surveillance to gain insights into lived experiences of the COVID-19 pandemic in Vietnam, Indonesia, and Nepal (all sites of Wellcome Africa Asia Program’s Oxford University Clinical Research Unit [OUCRU]) [33]. A total of 3 surveys, 2 in phase 1 and 1 in phase 2, were conducted in 13 districts in a range of urban, rural, and remote settings across the 3 countries.

Phase 1 aimed to explore the experiences and impact of COVID-19 on health care workers, health-related staff, and vulnerable communities.

Phase 2 aimed to explore themes around the acceptance and accessibility of vaccines.

In phase 1, health care workers (HCWs) and health-related staff in clinical and community settings were targeted, including physicians, nurses, pharmacists, laboratory scientists, community health workers, ambulance drivers, cleaners, administration staff, and other health professionals. In the phase 1 community survey and phase 2 vaccine survey, general populations and vulnerable communities were targeted. These included specific populations, such as people in quarantine areas, recovered patients, older adults, new mothers, tuberculosis and patients with hepatitis C, and ethnic minority groups.

Throughout the project, communications between the teams at all 3 sites (Vietnam, Nepal, and Indonesia) occurred weekly through Zoom (Zoom Communications). This involved discussions around the initial project setup, development of data collection forms and processes, and data quality checks.

### CoAct Project

The CoAct project is also a mixed methods study to evaluate the impact of educational and participatory learning and action interventions in communities on antibiotic prescribing, antibiotic use, and antibiotic resistance. A baseline household survey was conducted in 64 rural communes from 3 districts in Nam Dinh province, northern Vietnam—Nghia Hung, Xuan Truong, and Hai Hau. The target population was a representative sample of the normal residents of 64 communes who were willing and able to give informed consent for participation.

## Ethics Approval

Both studies received institutional review board approvals from the Oxford Tropical Research Ethics Committee (OxTREC), as well as the National Hospital for Tropical Diseases Ethics Committee (Hanoi, Vietnam), Hospital for Tropical Diseases Ethics Committee (Ho Chi Minh City, Vietnam), Ethics Committee of Nepal Health Research Council (Kathmandu, Nepal), Patan Hospital Ethics Committee (Kathmandu, Nepal), and Ethics Committee of the Faculty of Medicine, University of Indonesia (Jakarta, Indonesia). In addition, we obtained local government permission as required by the local regulations in each project. Participants were informed about the study, and their participation was voluntary. For online surveys, participants must agree to participate before accessing questions; for remote interviews, consent is obtained through email or verbally recorded if email is unavailable. The decision to participate or not had no consequences on the participant’s benefits. All data are securely stored on an encrypted OUCRU server, with identifying information kept separate from survey responses, media anonymized or destroyed if consent is not given, and anonymous qualitative and quantitative data available upon reasonable request. For SPEAR, participants who were recruited directly were reimbursed for their participation and received $100,000 VND (US $3.83). Participants who were recruited through links shared on social media were not reimbursed. Participants who completed the CoAct household survey received $50,000 VND (US $1.91).

## Project Design and Management Framework

### Tool/Survey Development

For both studies, we implemented our research data collection using REDCap to provide security, privacy, and confidentiality to all participants. We set up REDCap for the two projects on a dedicated REDCap server in Hanoi, Vietnam; thus, all data was received in a central location. For each study, we established a working group to design the REDCap data collection forms, test, pilot, update, and review changes.

Our standard questionnaires for the 3 SPEAR surveys were designed with country-specific adaptations and translated into local languages by research teams. We used the multilingual hook package in REDCap for translating every variable into 4 languages (English, Nepali, Vietnamese, and Bahasa) without the need to create separate surveys for each country. The translations were then reviewed to ensure the surveys were correctly adapted after being designed in REDCap. For the CoAct study, the entire survey was conducted in Vietnamese. The data collection tool consisted of eight instruments covering general and specific information (demographic, socioeconomic, social capital, knowledge of antibiotics and antibiotic resistance, birth history, immunization, illness, and treatment). Instruments two (household member), six (child nutrition and vaccination history), and seven (household member illnesses) were repeat instruments for entering multiple members in a household.

The surveys were tested and piloted at all sites, and changes were implemented before the initiation of data collection. Data access groups (DAGs) were set up to organize the data for participating sites for the SPEAR project. In that way, local researchers could only work on and access the project data from their site, which facilitated subsequent data management. For CoAct, we did not have DAGs because the data was collected in one location, though a variable was included to identify districts. After revisions and updates were finished, surveys were moved to “production mode,” and the data collection period started.

### Data Collection and Deployment

#### SPEAR

Data collection for phase 1 was between June 2020 and December 2021. Data collection for phase 2 was between September 2021 and August 2022. Some surveys were self-completed, where participants had access to an electronic device and the internet. However, some interviews were conducted in person or over the phone, where participants were unable to self-complete the survey due to low digital literacy or unreliable local internet access. The Mode of data collection also depended on the public health restrictions in place in the area at the time. Information about the study was provided on the first page, and participants were asked to click a link stating that they agreed to participate, which allowed access to the survey questions.

#### Phase 1

Links to web-based surveys and QR codes were posted on institutional websites and shared through professional networks and social media channels, including Facebook and institutional websites, in Nepal and Vietnam, to reach as wide an audience as possible. We sought permission to contact discharged patients with COVID-19 and people within or discharged from quarantine centers, in the hospital sites managing patients, and quarantine centers. These participants were contacted by phone. We used our extensive networks within the health care systems and communities in each country to identify participants without internet access to take part in telephone interviews. For targeted health care workers interviews, we randomly sampled participants from staff lists at selected hospitals and health centers linked to partner institutions using computer-generated random numbers stratified by department and sex. For targeted community interviews, we used randomly sampled participants from household listings obtained from our local community-level partners, where possible, as well as purposive sampling.

#### Phase 2

We first targeted participants from the community survey in phase 1 for whom we had a contact phone number or email address. We then randomly selected additional participants from household lists using computer-generated random numbers, in order to reach our minimum sample size for each setting (400 per country per survey). We also posted links to the survey web-based to be shared through social media, including Facebook, institutional websites, and professional networks.

#### CoAct

The survey was a cross-sectional household survey forming the baseline of a cluster-randomized trial. Data collection was completed district by district: Nghia Hung in April 2022, Hai Hau in April 2023, and Xuan Truong in May 2023. A list of communes with active Community Health Centers was compiled by province-level officials. A total of 64 communes meeting eligibility criteria were chosen for the study. In each community, we randomly sampled approximately 195 people for each survey. Assuming 10% refusal, we had 175 per cluster and 11,200 in total. One adult household member who was identified as the main caregiver was invited to answer for all other household members. We trained 64 local interviewers, who were mainly community health workers. They used the web-based version of REDCap on internet tablets to interview and support participants throughout the interviewing process. Some data collectors used paper questionnaires as they were unfamiliar with digital technology. We first created a supplementary screening survey to obtain participants’ consent from every selected household before implementing the main survey. Unlike the SPEAR survey, for some participants, the CoAct survey was not completed at one time. For households with children under five, data collectors needed to complete the first part of the questionnaire on the first visit and then finish the questionnaire when they came back to collect children’s samples.

### Data Storage and Management

For SPEAR participants who filled in the survey on the web, their responses were routed directly into a REDCap server hosted by OUCRU. For participants who completed a phone or interview-administered survey, responses were entered into REDCap directly (if the interviewers had access to the internet) or documented on paper and then entered into REDCap from the paper form. All laptops and devices used for data collection and data storage were encrypted and password protected. Email addresses and phone numbers for future contact and linkage were stored separately from the survey responses.

CoAct data were collected using internet tablets, and data were entered using the REDCap web platform. Data were uploaded immediately to a secure REDCap server hosted by OUCRU. All devices used for data collection and storage were encrypted and password-protected. Data monitoring and quality control measures were implemented in real time using a collaborative approach. Every week, our data manager at OUCRU Hanoi runs an automated check (type of variables, length of the information, range of values, duplication records, coherence between information, etc) by using the Data Exports, Reports, and Stats feature to make the survey reports for quality control purposes. When an issue was identified, the data manager raised a query and notified the site coordinator. Data quality reports were updated whenever new issues for monitoring and checking were identified.

**Figure 1:**
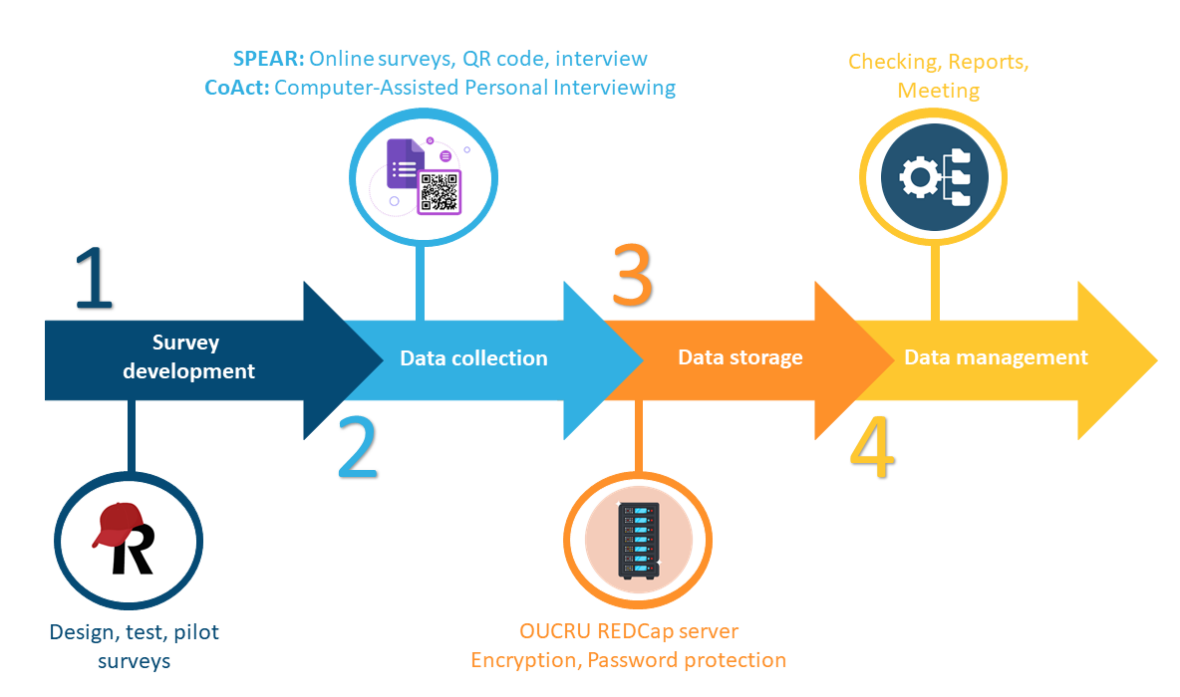
Data pipeline overview: tools, decisions, and stages. CoAct: Collective Action against antimicrobial resistance in Vietnam; OUCRU: Oxford University Clinical Research Unit; REDCap: Research Electronic Data Capture; SPEAR: Social Science, Public Engagement and Action Research.

The field-site data collection coordinator regularly used the automated reports and arranged a meeting or contacted the data collection team to resolve queries and provide missing or corrected data. Queries were later reviewed and closed by the data manager. In case problems were experienced by interviewers during data collection, these were reported to site coordinators and data managers for resolution. Weekly data backups were performed to maintain up-to-date datasets. Figure 1 provides an overview summarizing all key stages conducted in the two projects, highlighting the tools and methods used to enhance the clarity and accessibility of the methodology.

### Data Collection Results

Timely completion of data collection was a key success, with 95% of data collection completed within the project timeline. As of December 2022, the SPEAR study had completed data collection across 13 sites in three countries. A total of 3 surveys were completed, and data were obtained. In phase 1, after data cleaning, the community survey collected 1825 records overall, including 809 records from Indonesia, 504 records from Nepal, and 512 records from Vietnam. Meanwhile, the health care worker survey collected 2321 in total, with 487 Indonesian records, 537 Nepal records, and 1297 Vietnam records. The data completion rate for the community and health care workers survey was high, with around 80% of records deemed complete and usable for analysis. In phase 2, a total of 1915 records were collected, of which 891 were from Indonesia, 405 from Nepal, and 619 from Vietnam. The completion rate for this phase reached 85%, reflecting efficient data collection procedures and quality control efforts. In the CoAct study, our REDCap system was used to collect data from 3237 households in Hai Hau, Nghia Hung, and Xuan Truong districts in Nam Dinh province. The study achieved a completion rate of 100%, with all households providing fully completed records, contributing to the robust and reliable dataset for analysis.

### Challenges and Solutions

We encountered several challenges using REDCap for data collection. Some of the issues were shared between the two surveys, others were unique to each study. Problems and proposed solutions are summarized in Table 1.

**Table 1:** Challenges and solutions for REDCap (Research Electronic Data Capture) electronic data capture during Social Science, Public Engagement and Action Research study (SPEAR) and Collective Action against antimicrobial resistance in Vietnam (CoAct) survey implementation.

Challenges		Solutions
**Tool development**		
	**For SPEAR**	
	Setting the multi-language tool with different answer options for some country-specific questions led to branching logic conflicts.	Participants selected their language at the beginning of the survey. Different country-specific responses were recorded in separate variables, which were merged during data processing. Careful piloting in each language/site before moving to Production mode to minimize the need for later revisions.
	Developing the tools and making changes was time-consuming due to the complexity of questionnaires and the separate time zones across three sites.	We held weekly Zoom meetings across three countries to share feedback from survey development, testing, and piloting.
	**For CoAct**	
	A lengthy questionnaire with complex logical connections between instruments required back-and-forth testing between survey creators and community data collectors.	Careful piloting, training, close supervision, and regular, routine data checks to ensure completeness and logical responses.
	REDCap does not allow data to be collected with relational data structure for nested forms, eg, members within households. Unique primary identifiers (keys) are automatically created as record IDs, but they are difficult and meaningless for data collectors to work with.	We created separate REDCap instruments relating to nested forms (households, household members, children, illness episodes). These could be identified by Record ID, instrument ID, and instance ID. So that data collectors could select a specific household member in nested forms, we created a separate field and used an SQL command to display member household names in a dropdown list, which they could choose from. This meant that data collectors did not need to reenter the member’s name to link the records, thus avoiding typing errors.
	To reduce survey time, we wanted to randomly present only two questions out of a set of seven questions to each household.	We created a random combination of questions for each household by group outside of REDCap, then we used branching logic to show only the questions that were pre-assigned for that household.
**Data collection**		
	**For SPEAR**	
	Some real data was mistakenly entered before production mode, and this made data cleaning time-consuming.	We downloaded the data and reimported it to REDCap once production mode had started. We regularly scheduled communications between the teams at all three sites to discuss the initial project setup, data quality checks, and verification of data collection. Future efforts should be clear in communication with team members when making the transition to production mode.
	Incomplete and duplicate records due to internet disruption during data collection, ie, respondents started the survey, got cut off, and started again with a new, duplicate record.	We added a time stamp and field to indicate when the survey was actually completed, rather than cut off midway.
	**For CoAct**	
	Some data collectors were not comfortable using electronic devices and opted to use paper surveys, which were entered into REDCap later.	Carefully planned REDCap training to provide basic working knowledge and hands-on experience using the software, ensuring enough training time and user-friendly materials. The option for dual modes of data collection was made possible. Supervisors checked and entered paper surveys into REDCap.
	The mobile app had some disadvantages: (1) the app could display survey questions in Vietnamese, but some buttons, options, and system messages could only be displayed in English; (2) changes to the questionnaire were updated instantly on the web version but data collectors had to manually update the app on their device; and (3) data was not uploaded immediately and automatically onto the server, data collectors would have to upload manually from the app on their device.	Given that all study areas had mobile network coverage, and many data collectors lacked digital literacy skills, we decided to use the web-based version instead of the mobile app.
	Some data needed to be collected at a second visit, but data collectors lacked the digital literacy required to easily navigate and find records in REDCap, and it would be complicated to manage permissions for 64 user accounts.	Data collectors could re-enter the survey later for the second visit by requesting supervisors to send survey links and return codes.
	Incomplete and duplicate records due to internet disruption during data collection, ie, respondents started the survey, got cut off, and started again with a new, duplicate record.	If data collectors were cut off during the interview, they had to ask the supervisor to send a link to re-enter the survey. We created automated REDCap reports to display all records entered for each commune, so that supervisors could use these to monitor data collection progress and check for errors. Ongoing technical support and real-time data checking to identify and remove incomplete and duplicate records. We also added a time stamp and field to indicate when the survey was actually completed, rather than cut off midway.
	Data collectors lacked sufficient digital literacy to navigate the REDCap system or mobile app. To simplify the process, they entered REDCap through the webform, rather than logging in with an account. This meant that they could not directly re-enter or check records. Following internet disruption or to complete a later section, they had to request a link email to re-enter. This was confusing and complicated because it had to be done manually, case by case.	We developed a standardized data collection SOP to provide guidance for managing data and guaranteeing that all data was gathered, checked, and properly evaluated. Supervisors were trained to use the REDCap system to check data, identify records, and send survey links to data collectors. Strictly follow the data collection timeline in order to manage reports from different sites as new data is entered or edited.
	Some error messages could not be displayed in languages other than English.	We added footnotes or pop-ups for more information in the local language and to explain questions.
**Data management**		
	**For SPEAR**	
	Because many surveys were self-completed, we had difficulty distinguishing between incomplete surveys and surveys with a lot of skipped questions.	We used the variables for time-stamp and completed survey status to identify incomplete surveys. During data cleaning, we removed any remaining records with less than 50% of the total questions answered.
	**For CoAct**	
	Managing incomplete and duplicate records for some households.	We used the time stamp and field to identify completed surveys. Careful checking by supervisors and cleaning by the data team to remove incomplete and duplicate records after data collection.
	Missing and incorrect data were identified during automated quality control checks.	To improve the data integrity and accuracy, we designed the tools with real-time validation checks, such as ensuring numbers are within a specified range or text is properly formatted. So, error notification can be raised for inconsistent or missing data. Phone numbers were collected during data screening, and call-backs were made to 20% of households to check data quality. Phone calls were also made to check for any missing or incorrect data found through error reports. We collected GPS data from the tablets for location tracking and used this to identify data collectors who needed extra supervision to ensure interviews were done.
	Record ID was used instead of household number, leading to duplication within the same household when starting the survey.	An extra field was created to mark the duplicated household entries, which was reviewed weekly for correction by the data manager.
	REDCap has many advanced modules and functions, some of which require IT skills and time to solve.	We sought advice from the REDCap community to solve problems. For future research, dedicated IT support and data managers would improve the capacity to modify tools and reports efficiently.

### Advantages of Using REDCap

REDCap is a user-friendly software without requirements of knowing programing to set up a database or project. Implementation of REDCap data capture allowed our researchers to gather data easily. We were able to quickly obtain large, diverse datasets without having to travel across nations, which was a great advantage, especially during the COVID-19 pandemic. Our labor force requirements and financial expenditures for the data collection procedures were greatly lowered by this solution, because costs for printing and manual data checking and entry were not required. Surveys could be developed in multiple languages, including error messages. We were able to collaborate across multiple countries during remote working periods, and the flexibility of REDCap allowed us to make updates and corrections to problems identified in data collection tools even once data collection was in progress, without having to reprint many paper questionnaires.

The screening and consent processes were integrated with data collection, allowing for the collection and storage of metadata, including refusals. There are some projects discussing the development, piloting, and review of REDCap data collection forms in order to offer practical recommendations, then ensuring reliable and high-quality data collection [27,34]. Real-time monitoring, data checking, and correction were possible. Data collectors could check data immediately after collection, which minimized errors going unnoticed. It also provided easy exports, so users were in control of their data at any time. Additionally, REDCap automatically generated a codebook and data dictionary, which provided good documentation on the meaning of the variables and made collaborative work analyzing data easier. Fields in REDCap can be marked as identifiable, and the data manager has the option to de-identify the data during export. This function is extremely valuable for datasets including personal information such as name, address, and health insurance number, allowing us to share those datasets without being afraid of disclosing personally identifiable information. Table 2 summarizes the advantages and disadvantages of electronic data capture compared to paper-based data collection that were identified during the implementation of the two projects.

Use of REDCap has significantly increased in epidemiology, particularly in studies undertaken in low- and middle-income countries [35]. The key advantages of using REDCap software are the reduction of research costs and improved efficiency. There is no charge for REDCap for use by educational institutions, which is an advantage compared to some other software. REDCap provides its tools freely and has a lot of effective functions, which make global research collaboration efficient. Examples of efficiency have been reported in several cohort studies and large international collaborations, illustrating that there were reductions in time and costs associated with interviewing, traveling, printing documents, and communicating between sites [6,36,37]. As compared to paper-based research methods, these savings have led to a lower cost per interview and overall expenditure. Financial advantages increase with the size of the research population [38].

In our two studies, REDCap enabled us to collect data from large populations across a range of urban, rural, and remote settings. We were able to control data entry errors and increase data quality by using a range of functions in REDCap, including field validation, question requirements, and branching logic. Data inconsistencies in research tend to occur quite commonly for several reasons, mostly due to a frequent lack of protocol content and appropriate training and education of staff [39]. However, with the help of REDCap’s function for field restrictions, it was possible to minimize data recording errors when conducting interviews. In addition, branching logic could be applied so that, given a participant’s response to one question, data collectors would automatically be directed to the correct follow-up question or skip subsequent questions that were not relevant. Checking this branching logic took time during setup, but it was an important step to have a well-prepared survey, and it saved time later for error checking. Some errors or inconsistencies still arose, particularly in managing duplicate or incomplete records, which could lead to invalid or misleading findings, a reduced sample size for analysis, and incorrect conclusions To resolve these issues, we contacted the interviewee to verify the information given and applied statistical methods during the data manipulation process, such as calculating the percentage of completion based on the number of required questions answered. Only records with at least 80% completion were included in the final analysis. For validated scales (such as the 21-item Depression, Anxiety, Stress Scale), we used recommended methods for managing records with missing data [40,41]. With immediate access to electronic data, it was possible to automate error and consistency checking and identify problems. Research supervisors were thus able to identify errors during the data collection period and fix them soon after the interview. This contrasts with paper-based questionnaires, where it can take months to achieve a closed database since questionnaires must be gathered, checked manually, and entered.

Time-saving was another benefit of the use of electronic data capture. REDCap features, such as automated survey invitations and reminders, streamlined the process of reaching participants without the need for in-person contact. Additionally, its built-in data validation and real-time error checking reduced the need for extensive data cleaning, thereby speeding up the analysis phase. Our findings strongly support the idea that REDCap is a good choice for developing and deploying population-based data collection, especially during the COVID-19 period when conducting face-to-face interviews was difficult due to social distancing measures in place [42]. REDCap’s capabilities greatly enhanced the efficiency and effectiveness of our research efforts during the project implementation period. Overall, by increasing accessibility, inclusivity, data quality, and local ownership, REDCap can play a vital role in addressing disparities in global health research today. Integrating REDCap into research in Indonesia, Nepal, and Vietnam improved operational efficiency and ensured that disadvantaged and rural areas are not overlooked in intervention strategies. However, caution is needed to ensure that the lack of digital literacy in some settings does not pose a barrier to using REDCap for data collection and inadvertently perpetuates disparities in the availability of data to advance understanding of population health. In the CoAct study, all data were collected by interviewers, while in the SPEAR study, we complemented self-reported data collection with interviewer-led data collection to ensure that lack of digital literacy did not prevent us from capturing information from vulnerable groups like those in remote areas, with lower income, or older adults.

**Table 2:** Advantages and disadvantages of electronic data capture in comparison with paper-based data capture.

Paper-based data capture		Electronic data capture
**Data collection**		
	**Advantages**	
	No IT skills are needed to develop forms or use data collection tools. Data can be collected in various formats (typed, handwritten, etc.) and without the need for specific hardware, software, electricity, or reliable internet or technology infrastructure	Free data capture software is widely available, particularly for educational institutions, enabling time-saving data entry during collection. Field restrictions, branching logic, and mandatory fields avoid errors and reduce missing answers. Additional digital data can be collected, like audio recordings of verbal consent statements, GPS coordinates, and photos. Self-completed surveys provide links that enable large-scale deployment across sites and regions, lowering collection costs. Mobile apps offer flexible, offline data entry in remote areas.
	**Disadvantages**	
	High cost and resources for printing and distributing questionnaires. Data collectors may struggle with branching logic or record unrealistic values. Risk of missing data from illegible writing, lost forms, or incomplete responses.	Cost of devices (such as a tablet or smartphone) and trained IT personnel for form development and device maintenance Internet connectivity is required to transmit data, and electricity/internet limitations may restrict use in some areas. Basic IT skills are needed for participants or interviewers to enter data.
**Data management**		
	**Advantages**	
	Data entry errors can be verified by checking the original paper record.	Data can be collected and entered in real-time, ensuring timely activities tracking, reducing errors and missing data, and being immediately stored for verification. Data are easy to edit, update, or delete, with multilanguage support for default and additional languages. Many platforms offer built-in tools for viewing reports, visualization, advanced analytics, and remote collaboration for efficient data processing and sharing.
	**Disadvantages**	
	Time-consuming to enter and check large amounts of data. Data entry errors can occur and require additional human resources to minimize through checking or double-entry of data.	Data entry errors cannot be verified against the original record. Device damage or malfunction may cause data loss or corruption.
**Data storage**		
	**Advantages**	
	No technical skills required to access and check paper-based records.	Data is immediately stored, can be backed up to avoid loss, and is easy to search in digital records.
	**Disadvantages**	
	Storage of paper questionnaires can be bulky, especially for large surveys Well-maintained filing systems are needed to retrieve specific documents. Paper can easily be stolen, damaged, or destroyed with incorrect storage.	Sufficient data storage capacity may be needed for large datasets. Risk of data breaches or hacking can compromise data security.
**Security**		
	**Advantages**	
	Physical access to raw data storage units can easily be monitored. Unlikely that copies of physical documents will be inadvertently made and shared.	Access to data can be restricted from the point of data collection. Better security measures, including encryption and backup systems.
	**Disadvantages**	
	Difficult to ensure the security of paper questionnaires during transit. Once physical data is entered into a database management system, it may still be inadvertently copied and shared	Data may be inadvertently copied and shared.

### Limitations and Future Recommendations

We chose REDCap because of the relatively simple interface, meaning that it was easy to get started without specific programming skills. However, preparing the REDCap server for data collection and developing survey tools with multiple instruments and complex branching logic was not such a straightforward process. It required a professional with sufficient competence to install and configure the necessary tools and tailor the REDCap environment using some SQL programming. Although REDCap provides various useful features like real-time validation rules, required fields, audit trails, etc, errors such as incomplete and duplicate records can still happen, especially when data entry involves large teams or intricate workflows. These issues are one of the system's potential drawbacks, which call for specific solutions, such as routine data quality checks, iterative validation, and careful supervision of data collection. Thus, the project team needs to have an IT expert to develop and test the tools, as well as sufficient technical expertise among the supervisory team to troubleshoot, communicate issues to the IT expert, and manage data checking, editing, and correction within REDCap. This would speed up the data collection and verification processes. In populations with low digital literacy, thorough training, piloting, easy-to-follow guidance materials, and close supervision are needed.

A vital element of REDCap tool development and implementation is effective and close collaboration between sites implementing projects. Given the complexity of the 2 projects, such as managing multi-language translations and intricate branching logic, which REDCap alone could not fully address, we had to set up weekly meetings to facilitate back-and-forth discussions between sites. During the development period, these meetings focused on developing the tools, transferring them to REDCap, testing the system, piloting, and revision. Weekly meetings continued during implementation periods to identify and troubleshoot common problems and collect status updates. One frequently discussed issue was the occurrence of multilingual and branching logic errors, which appeared as notifications at the beginning of the web survey. Continuous feedback allowed data collectors to identify and report inaccuracies, enabling data managers to resolve them promptly. This iterative process of feedback and correction facilitated the enhancement of the tools and the overall approach. For both SPEAR and CoAct studies, our research involved collaboration with multiple partners and data collection across diverse locations. The entire data management process, which included gathering, storing, and monitoring data, was streamlined with REDCap. Some features were not straightforward for staff and participants with basic computer literacy skills. These problems could be rectified with carefully designed training, supervision, and dedicated IT support. More intensive piloting and supervision were needed to familiarize data collectors with the REDCap tool and troubleshoot problems that might have been needed with a paper-based survey, especially in the early phases of data collection. These findings highlight the potential of EDC platforms like REDCap to transform data collection methods in low- and middle-income countries. Future research may leverage these technologies to boost productivity, increase data quality, and facilitate large-scale population studies by tackling the issues of digital literacy and early adoption. Our results highlight how EDC platforms like REDCap may have a significant impact in low- and middle-income countries, specifically by tackling issues related to integrity in resource-limited settings. By detailing our lessons learned—such as the importance of iterative testing, regular inter-site meetings, and customized modifications—we provide a roadmap for future projects to boost productivity, enhance data quality, and effectively conduct large-scale population research. Textbox 1 provides a set of recommendations for potential future enhancements to REDCap, based on our experiences and identified areas for improvement.

**Suggestions for future Research Electronic Data Capture (REDCap) features.**

- The mobile app has modules to translate the interface into multiple languages; however, some buttons, options, and system messages can only be displayed in English. These also need to be translatable into local languages.
- Management of nested forms is complicated. More efficient management and storage of relational data structures would be beneficial for population-based surveys or longitudinal studies with repeated visits.
- REDCap automatically creates a record ID as the primary key. A secondary unique field can be added as a constraint to make sure that a record does not already exist. However, this must be a text field. To avoid typing errors, we wanted to use a drop-down list of preselected households from our sampling frame to check if a record for this household already existed in the database. However, it was not possible to set this non-text field as a secondary key.
- A codebook can automatically be created and downloaded; however, this is not currently in a table format. All values for a question are contained within one cell, rather than as separate rows.

## Abbreviations

CoAct: Collective Action against antimicrobial resistance in Vietnam
DAG: data access group
EDC: electronic data capture
FISMA: Federal Information Security Management Act
GDPR: General Data Protection Regulation
HCW: health care worker
HIPAA: Health Insurance Portability and Accountability Act
ODK: Open Data Kit
OUCRU: Oxford University Clinical Research Unit
REDCap: Research Electronic Data Capture
SPEAR: Social Science, Public Engagement and Action Research
